# Editorial: Micro-scale vegetable production: sprouts and microgreens as innovative functional foods

**DOI:** 10.3389/fpls.2024.1395888

**Published:** 2024-03-25

**Authors:** Martina Puccinelli, Fernando Malorgio, Massimiliano D’Imperio

**Affiliations:** ^1^ Department of Agriculture, Food and Environment, University of Pisa, Pisa, Italy; ^2^ Institute of Sciences of Food Production, National Research Council (CNR), Bari, Italy

**Keywords:** biofortification, substrate, mineral concentration, hydroponics, indoor cultivation

In recent years, sprouts and microgreens have gained popularity as innovative functional foods due to their attractive colors, textures, and flavors. Microgreens exhibit higher phytochemical and antioxidant content compared to mature leaves (Lone et al., 2024). Various factors including plant genotype, cultivation system, environmental conditions, and post-harvest storage affect phytochemical concentration in sprouts and microgreens (Teng et al., 2023). The main focus of this Research Topic was the study of microgreens, and how genetic and pre-harvest factors can influence agronomic and nutritional quality of microgreens.

Microgreens are also appreciated for their mineral content (Teng et al., 2023). The genetic variation of mineral content, comprised NO_3_, was evaluated by Di Gioia et al. in a study conducted on 17 microgreen species. A modified half-strength Hoagland solution, free of microelements, was used to fertigate microgreens after germination. Ten to 19 day-old microgreens (according to plant species) were harvested, and yield and mineral content were measured. The principal macronutrients present in microgreens were N and K. In general, the tested species showed a high NO_3_ content, higher than 1000 mg/kg fresh weight. Eight of the tested species represented a good dietary source of K. Some microgreens species were a good source of Ca and Fe: scallion, red cabbage, amaranth, and Genovese basil. A significant amount of Cu or Zn could be provided to the diet only by the consumption of scallion and shiso, or sunflower microgreens, respectively. The genotype strongly affects the growth and mineral concentration of microgreens, and their consumption with the diet could address several dietary needs. The biofortification of microgreens with micronutrients could be acquired by supplementary fertilization.

Despite the importance of biofortification to increase the nutraceutical quality of products, there is limited research focused on Zn biofortification of microgreens, and only few plant species have been investigated (Consentino et al., 2023). Poudel et al. studied the effects of Zn biofortification with different Zn sources (ZnSO_4_, Zn-EDTA, and ZnO) and concentrations (from 0 to 200 mg/L), by seed nutri-priming, on pea and sunflower microgreens yield and quality parameters. In both plant species, the highest Zn content was detected in microgreens treated with 200 mg/L of ZnSO_4_. Treatment with Zn-EDTA did not induce Zn accumulation in microgreens and did not affect their quality characteristics. Conversely, compared to Zn-EDTA, ZnO application induced higher chlorophyll and total phenols content as well as higher antioxidant capacity in microgreens. Moreover, soaking seeds in solution containing high concentrations of ZnSO_4_ and ZnO reduced the phytic acid/Zn molar ratio, in microgreens of both species, increasing the bioaccessibility of Zn. The positive effect on quality parameters, and Zn content of microgreens, make the seed priming a feasible technique for microgreen Zn biofortification. The Zn chemical form, the plant species and the desired Zn microgreen concentration have to be taken into account to choose the optimal Zn dose.

Microgreen yield and quality, and their production sustainability, are also affected by the growing media (Lone et al., 2024). The effects on yield, mineral concentration and quality parameters, of different substrates were evaluated by Poudel et al., in a study conducted on peas and radish microgreens. The following substrates were tested: standard peat and perlite mixture (PP), coconut coir (CC), spent mushroom compost (SMC), organic waste compost (CMP), and 50:50 (v:v) mixes of PP and SMC, PP and CMP, CC and SMC, and CC and CMP. The high electrical conductivity of SMC strongly reduced the germination of seeds. The use of PP+CMP (50:50) mix led to a better quality of microgreens of both species, and to a higher or slightly lower yield of radish and peas, respectively. The possibility to replace part of PP with locally available CMP would reduce the cost and improve the sustainability of production process.

It is also possible produce microgreens without substrate. The growth and quality of five radish microgreens cultivars were evaluated when grown indoor, without substrate, using only H_2_O for irrigation (Tilahun et al.). All the cultivars adapted well to the cultivation system, and in 10 days their biomass increased from 6 to 10 times. The “Asia red” selection showed the highest yield, chlorophyll and vitamin C concentration, while the lowest yield was showed by “Koreon red”. “Asia purple” and “Koregon red” showed high dry biomass production, antioxidant capacity and content of phenols, flavonoids, anthocyanins, essential and total amino acids.

The possibility of being cultivated without substrate, their small size and fast growth, make microgreens suitable for production where commercial substrate, time, energy and volume are limiting factors, such as in Space (Teng et al., 2023), where plants can act as biological regenerators of air and water, by recycling consumables and waste while producing foods. The selection of suitable plant species for the production of microgreens in Space is crucial to obtain the best productivity and phytonutrient profile. An algorithm was applied by Izzo et al. to compare several plant genotypes grown as microgreens. The first selection was made using the data reported in literature. Twenty-five parameters related to the growth and concentration of phytonutrients were used. A mathematical model was employed to create a ranking of microgreen genotypes based on prioritization criteria. Subsequently, the six genotypes highest in the ranking were evaluated by cultivation. Moreover, the algorithm can be used to compare new species by combining parameters, which can be modified or re-prioritizing for specific selection purposes.

The development of indoor cultivation increases the interest in microgreens, which allow the quickly production, in a small space and with low inputs, of a product with high nutraceutical and commercial value. More research could be conducted to further improve the production and quality of microgreens, the response to post-harvest and the sustainability of the production process.

## Author contributions

MP: Writing – original draft, Writing – review & editing. FM: Writing – review & editing. MD’I: Writing – original draft, Writing – review & editing.
